# Magnesia in Poisoning by Arsenic

**Published:** 1854-02

**Authors:** 


### MAGNESIA IN POISONING BY ARSENIC.

An interesting case is related by Dr. Thomas R. H. Thomson, in a late number of the *London Lancet*, in which a woman took, by mistake, two scruples of arsenious acid. She was relieved by recently prepared hydrate of magnesia, of which not less than eight ounces were administered in the course of a few hours. The advantages of this antidote are, that it is perfectly safe, easy of preparation, and mixed with facility. As very little of the poison, in this case was detected in the urine, the presumption is that it was rendered inert by its combination with the magnesia, and escaped by the bowels.

